# Contrasting Transmission Dynamics of Co-endemic *Plasmodium vivax* and *P*. *falciparum*: Implications for Malaria Control and Elimination

**DOI:** 10.1371/journal.pntd.0003739

**Published:** 2015-05-07

**Authors:** Rintis Noviyanti, Farah Coutrier, Retno A. S. Utami, Hidayat Trimarsanto, Yusrifar K. Tirta, Leily Trianty, Andreas Kusuma, Inge Sutanto, Ayleen Kosasih, Rita Kusriastuti, William A. Hawley, Ferdinand Laihad, Neil Lobo, Jutta Marfurt, Taane G. Clark, Ric N. Price, Sarah Auburn

**Affiliations:** 1 Eijkman Institute for Molecular Biology, Jakarta Pusat, Indonesia; 2 The Ministry of Research and Technology (RISTEK), Jakarta Pusat, Indonesia; 3 Agency for Assessment and Application of Technology, Jakarta, Indonesia; 4 Faculty of Medicine, University of Indonesia, Jakarta Pusat, Indonesia; 5 National Malaria Control Program, Ministry of Health, Jakarta, Indonesia; 6 United Nations Children’s Fund (UNICEF), Jakarta, Jakarta, Indonesia; 7 Eck Institute for Global Health, University of Notre Dame, Notre Dame, Indiana, United States of America; 8 Global and Tropical Health Division, Menzies School of Health Research and Charles Darwin University, Darwin, Northern Territory, Australia; 9 Faculty of Infectious and Tropical Diseases, London School of Hygiene and Tropical Medicine, London, United Kingdom; 10 Faculty of Epidemiology and Population Health, London School of Hygiene and Tropical Medicine, London, United Kingdom; 11 Centre for Tropical Medicine, Nuffield Department of Clinical Medicine, University of Oxford, Oxford, United Kingdom; Barcelona Centre for International Health Research (CRESIB) and Institució Catalana de Recerca i Estudis Avançats (ICREA), SPAIN

## Abstract

**Background:**

Outside of Africa, *P*. *falciparum* and *P*. *vivax* usually coexist. In such co-endemic regions, successful malaria control programs have a greater impact on reducing falciparum malaria, resulting in *P*. *vivax* becoming the predominant species of infection. Adding to the challenges of elimination, the dormant liver stage complicates efforts to monitor the impact of ongoing interventions against *P*. *vivax*. We investigated molecular approaches to inform the respective transmission dynamics of *P*. *falciparum* and *P*. *vivax* and how these could help to prioritize public health interventions.

**Methodology/ Principal Findings:**

Genotype data generated at 8 and 9 microsatellite loci were analysed in 168 *P*. *falciparum* and 166 *P*. *vivax* isolates, respectively, from four co-endemic sites in Indonesia (Bangka, Kalimantan, Sumba and West Timor). Measures of diversity, linkage disequilibrium (LD) and population structure were used to gauge the transmission dynamics of each species in each setting. Marked differences were observed in the diversity and population structure of *P*. *vivax* versus *P*. *falciparum*. In Bangka, Kalimantan and Timor, *P*. *falciparum* diversity was low, and LD patterns were consistent with unstable, epidemic transmission, amenable to targeted intervention. In contrast, *P*. *vivax* diversity was higher and transmission appeared more stable. Population differentiation was lower in *P*. *vivax* versus *P*. *falciparum*, suggesting that the hypnozoite reservoir might play an important role in sustaining local transmission and facilitating the spread of *P*. *vivax* infections in different endemic settings. *P*. *vivax* polyclonality varied with local endemicity, demonstrating potential utility in informing on transmission intensity in this species.

**Conclusions/ Significance:**

Molecular approaches can provide important information on malaria transmission that is not readily available from traditional epidemiological measures. Elucidation of the transmission dynamics circulating in a given setting will have a major role in prioritising malaria control strategies, particularly against the relatively neglected non-falciparum species.

## Author Summary

The malaria parasite *Plasmodium vivax* is a growing public health burden across the globe. Largely overshadowed by the more fatal *P*. *falciparum* parasite, increasing reports of anti-malarial drug resistance and life-threatening disease complications demand concerted efforts to eliminate *P*. *vivax*. Outside of Africa, *P*. *vivax* usually co-exists with *P*. *falciparum*. In these regions, malaria interventions have demonstrated greater success against falciparum. The authors genotyped *P*. *vivax* and *P*. *falciparum* parasites from 4 co-endemic sites in Indonesia, and used the data to gauge how frequently and how focally parasites were transmitted, and how much they spread between sites. Their results indicated that whilst the *P*. *falciparum* populations displayed evidence of low and unstable transmission, the *P*. *vivax* populations were more diverse and exhibited more stable transmission patterns, requiring different intervention approaches. Relative to *P*. *falciparum*, the *P*. *vivax* parasites demonstrated evidence of greater spread between populations, possibly facilitated by the dormant liver stage which enables *P*. *vivax* to be carried asymptomatically in patients for long periods of time. These findings highlight the need to reconcile the intervention requirements for non-falciparum malaria species in co-endemic regions, and the general utility of parasite genotyping to facilitate surveillance.

## Introduction

*Plasmodium vivax* is a major global health burden, with an estimated 2.85 billion people living at risk of infection [1]. Rising levels of multidrug resistance and severe disease [2–7], and the demonstrated transmission amongst Duffy blood group-negative individuals [8–12], demand concerted efforts to control and eliminate the parasite. However, relative to the other major human malaria pathogen, *P*. *falciparum*, interventions against *P*. *vivax* have been less effective [13]. Critical to the success of control and elimination programs is the acquisition of data on the intensity and dynamics of local malaria transmission. Measures of transmission intensity have been used to inform public health interventions for *P*. *falciparum* [14], however the dormant hypnozoite stage of *P*. *vivax* result in its dynamics being far more challenging to interpret. Outside of Africa, *P*. *vivax* usually co-exists with *P*. *falciparum*. In these co-endemic regions, potential differences in the respective transmission of the two species further complicate decision-making on the prioritization of intervention activities.

Previous studies have demonstrated the utility of genotyping parasite population samples to inform on *P*. *vivax* and *P*. *falciparum* diversity, population structure and underlying transmission patterns [15–35]. These molecular approaches complement the more traditional measures of transmission intensity. As well as providing an independent gauge of intensity, parasite genotyping can inform on other features of transmission such as outbreak dynamics, infection spread within and across borders, and reservoirs of infection [36]. However few studies have demonstrated the utility of genotyping to compare the transmission dynamics of co-endemic *P*. *vivax* and *P*. *falciparum* isolates [18,19,28]. A combination of the widely contrasting malaria epidemiology and the physical geography of the Indonesian archipelago rendered this an ideal setting within which to further address the comparative patterns of transmission in *P*. *vivax* and *P*. *falciparum* populations in multiple co-endemic sites.

Malaria remains a major public health burden in Indonesia, with over 400 thousand confirmed cases in 2012 representing just a fraction of the actual suspected burden [37]. Malaria caused by *P*. *vivax* infection is a particular concern in Indonesia owing to the occurrence of high-grade chloroquine-resistant infections [38,39]. In 2009, the Ministry of Health instigated a malaria control and elimination strategy to be rolled out island by island with the aim of achieving nation-wide malaria elimination by 2030. One of the greatest challenges in achieving this target is the extensive heterogeneity in malaria epidemiology across an archipelago comprising more than 6,000 inhabited islands. The country is characterised by marked variation in the incidence of malaria, distribution of *Anopheles* vectors, prevalence of anti-malarial drug resistance, and ethnic, cultural and socio-economic diversity [40]. Malaria caused by *P*. *knowlesi* is also an emerging problem in Indonesia. However, until recently, the burden of *P*. *knowlesi* malaria has not been comprehensively investigated in Indonesia, with only two published studies confirming the presence of this species [41,42]. Hence interventions need to be tailored to the epidemiological setting in question. Furthermore, the highly mobile population increases the risk of re-introduction of malaria to areas with no malaria transmission.

As a prerequisite to developing a comprehensive national surveillance for malaria transmission, we used simple genotyping methods to characterize the genetic diversity and structure of *P*. *vivax* and *P*. *falciparum* isolates collected between 2011 and 2013 at four Indonesian sites with differing endemicity. The resultant insights into the local *P*. *vivax* versus *P*. *falciparum* transmission dynamics are reviewed and interpreted in the context of the country’s malaria elimination goals. The broader implications for prioritization of intervention strategies in other regions of the globe where the prevalence of non-falciparum malaria is rising are also discussed.

## Materials and Methods

### Study Sites and Sample Collection

**Fig 1:**
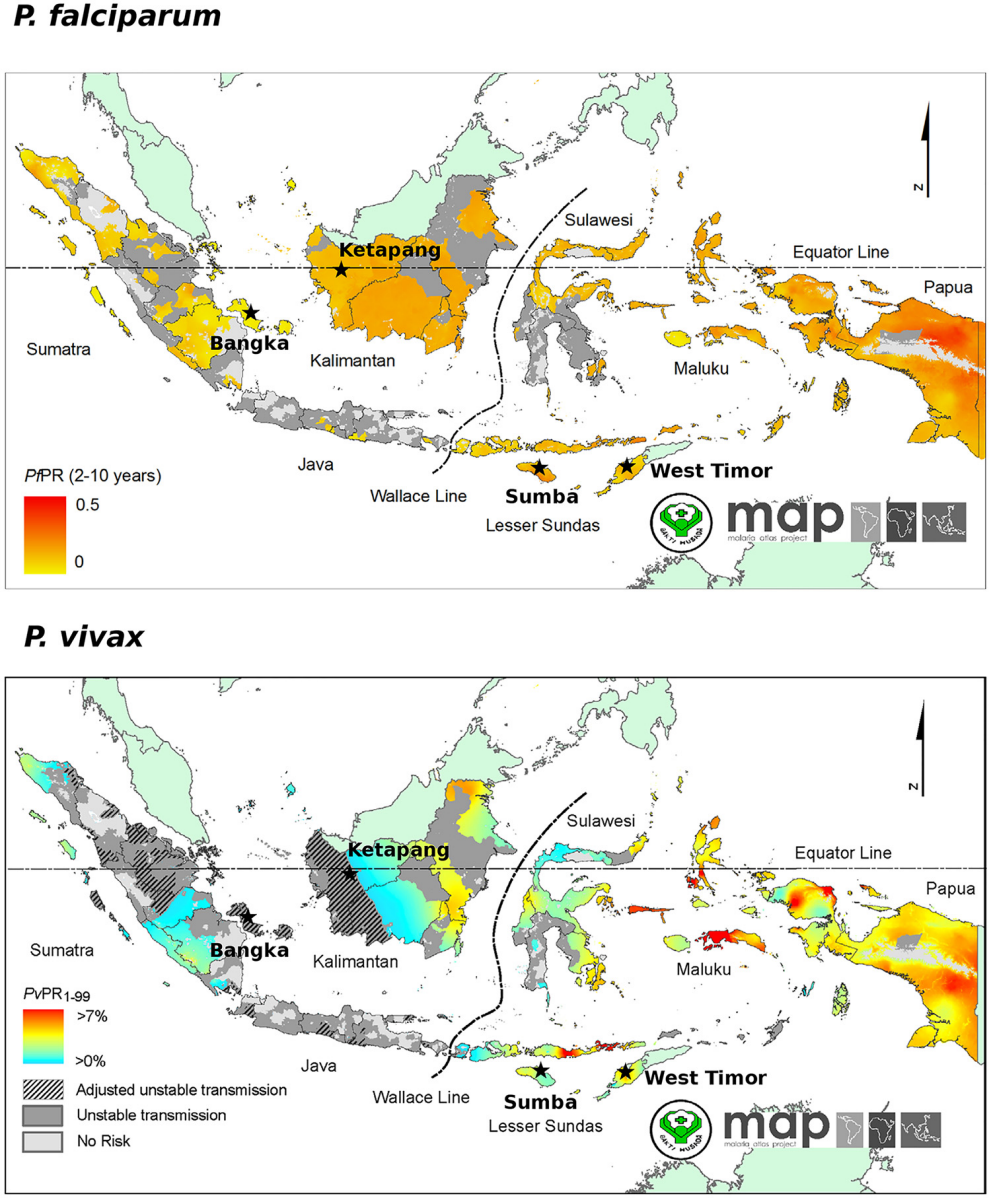
Prevalence maps. These maps were generated by the Malaria Atlas Project, University of Oxford. The colour scales reflect the model-based geostatistical point estimates of the annual mean *P*. *falciparum* parasite rate in the 2–10 year age group (*Pf*PR_2–10_) (top) [45] and *P*. *vivax* parasite rate in the 1–99 year age range (*Pv*PR_1–99_) (bottom) [46] within the stable spatial limits of transmission in 2010. The approximate locations of the study sites described here are indicated with black stars.

The study was conducted in two sites on Bangka island (Bangka-Belitung, Sumatra), one site in Ketapang Province in West Kalimantan, two sites in Sumba (East Nusa Tenggara) and one site in West Timor (East Nusa Tenggara) (Fig 1). Details on the study sites are provided in S1 Table. Bangka, Sumba and West Timor are defined as moderate stable endemic or ‘hypo-mesoendemic’, and the Kalimantan site as unstable endemic [14]. Whilst Bangka and Ketapang have been assigned goals of elimination by 2015, the goals for Sumba and West Timor are to reach pre-elimination by 2020, with national malaria elimination by 2030. In all four study sites, dihydroartemisinin—piperaquine is used as the primary treatment for both *P*. *vivax* and *P*. *falciparum* malaria as recommended by the Ministry of Health since 2008. As well as malaria incidence, the study sites exhibit several socio-economic and ecological differences reflective of the heterogeneity generally found across the Indonesian archipelago. Whilst Bangka is the largest producer of tin in Indonesia, in Ketapang the main industries are palm oil and rubber production and logging. In East Nusa Tenggara the main economy is subsistence agriculture, with Sumba being one of the poorest islands in Indonesia. The available information on vector species demonstrates heterogeneity across the study sites [43]. Details on the bionomics of each species can be found on the Malaria Atlas Project website [44]. Briefly, in West and Central Bangka, the dominant malaria vectors appear to be *Anopheles leucosphyrus*, *An*. *latens* and *An*. *sundaicus sensu lato*. All three species/species complexes can transmit both *P*. *vivax* and *P*. *falciparum* and tend to bite in the evening/night. Both endophilic and exophilic feeding behaviour has been demonstrated in *An*. *sundaicus*. *s*.*l*. and *An*. *leucosphyrus*. Whilst *An*. *sundaicus*. *s*.*l*. display varying degrees of indoor and outdoor resting, *An*. *leucosphyrus* and *An*. *latens* do not tend to rest indoors in the day. In Ketapang Province, *An*. *balabacensis*, *An*. *leucosphyrus* and *An*. *latens* are prevalent. *An*. *barbirostris s*.*l*. is also observed but likely to play little if any role in malaria transmission. *An*. *balabacensis* can transmit both *P*. *vivax* and *P*. *falciparum*, is largely exophagic, feeding in the evening/night. In East Nusa Tenaggarra, *An*. *sundaicus s*.*l*., *An*. *balabacensis* and *An*. *flavirostis* are prevalent. *An*. *flavirostis* is primarily zoophilic, displaying both endophagic and exophagic feeding in the evening/night. The vectorial capacity of this species in Indonesia remains unclear.

In Kalimantan, Bangka and Sumba, parasite DNA was collected by both active and passive detection of malaria-infected individuals, whilst in West Timor only active case detection was undertaken (Table 1). Passive case detection involved symptomatic patients presenting to outpatient health clinics in West Bangka, a hospital in South West Sumba, and West Kalimantan. At these locations, local policy stipulates that all malaria treatment should be based on microscopic screening of thick blood films. Active case detection was performed either through community surveys or through screening of individuals with fever.

**Table 1:** Details of parasite sampling.

Site	Species	Sampling	Collection period	No. patients	Median age, years	% male patients	Median parasite density, ul^-1^
Bangka	*P*. *falciparum*	Active	Oct 2011	22	25 (5–72)	59% (13/22)	1,346 (103–31,010)
		Passive	Oct 2011	36	30 (3–50)^1^	89% (32/36)	20,430 (198–755,400)^2^
		All	Oct 2011	58	30 (3–72)^1^	76% (45/58)	5,671 (103–755,400)^2^
	*P*. *vivax*	Active	Oct 2011	37	25 (1–45)	65% (24/37)	1,176 (69–87,770) ^3^
		Passive	Oct 2011	49	27 (3–50)	92% (45/49)	5,923 (73–529,600) ^1^
		All	Oct 2011	86	25 (1–50)	80% (69/86)	3,083 (69–529,600) ^4^
Ketapang	*P*. *falciparum*	Active	Oct 2012	3	7 (1–37)	33% (1/3)	392 (314–5187)
		Passive	Nov 2012-Jul 2013	10	21 (17–57)	90% (9/10)	11,380 (391–36,240)
		All	Oct 2012-Jul 2013	13	20 (1–57)	77% (10/13)	5,187 (314–36,240)
	*P*. *vivax*	Active	Oct 2012	2	10 (8–12)	50% (1/2)	402 (115–688)
		Passive	Oct 2012-May 2013	11	18 (9–43)	64% (7/11)	3,746 (759–21,670)
		All	Oct 2012-May 2013	13	17 (8–43)	62% (8/13)	2,589 (115–21,670)
Sumba	*P*. *falciparum*	Active	Jan-Nov 2012	45	9 (3–65)	53% (34/45)	4,052 (96–446,400) ^2^
		Passive	Jan-Feb 2012	15	15 (1–54)	53% (8/15)	38,370 (7,080–748,700) ^5^
		All	Jan-Nov 2012	60	10 (1–65)	53% (32/60)	7,080 (96–748,700) ^7^
	*P*. *vivax*	Active	Jan-Nov 2012	31	5 (0–30)	55% (17/31)	1,290 (69–20,050)^2^
		Passive	Jan-Sep 2012	9	30 (5–54)	78% (7/9)	9,923 (5,980–64,230) ^2^
		All	Jan-Nov 2012	40	5.5 (0–54)	60% (24/40)	4,091 (69–64,230) ^4^
West Timor	*P*. *falciparum*	Active	Jun-Jul 2013	35	16 (2–67)	60% (21/35)	480 (32–20,200)
	*P*. *vivax*	Active	Jun 2013	29	6 (1–37)	45% (13/29)	840 (48–11,000)
**All**	***P*. *falciparum***	**All**	**Oct 2011-Jul 2013**	**166**	**18 (1–72)** ^1^	**65% (108/166)**	**3,099 (32–755,400)** ^**9**^
	***P*. *vivax***	**All**	**Oct 2011-Jun 2013**	**168**	**18 (0.5–54)**	**68% (114/168)**	**2,110 (48–529,600)** ^**8**^

^1–9^ Superscript indicates the number of patients with missing data.

Following informed consent of the patient or their guardian, individuals with *P*. *falciparum* or *P*. *vivax* parasitaemia detected by blood film examination were asked to donate a venous (~1–2 ml) or capillary (~250 ul) blood sample collected into an EDTA-coated Vacutainer or microtainer or an anticoagulant-free 0.5 ml Eppendorf tube. Blood samples were stored at 2–8°C for up to 2 weeks, and -80°C for longer-term storage.

### DNA Extraction and Species Confirmation

DNA extraction was undertaken using the QIAamp blood mini kit (Qiagen) according to the manufacturer’s protocol. *Plasmodium* spp. was confirmed by PCR using the method described by Snounou *et al*. [47] and Boonma *et al*. [48].

### Genotyping

*P*. *falciparum* genotyping was undertaken at nine previously described short tandem repeat (STR) markers including *ARAII*, *PfPK2*, *poly-alpha*, *TA1*, *TA42*, *TA60*,*TA81*, *TA87* and *TA109*, using the primers and PCR conditions described by Anderson and colleagues [49].

*P*. *vivax* genotyping was undertaken at nine previously described STR markers including *Pv3*.*27*, *msp1F3*, *MS1*, *MS5*, *MS8*, *MS10*, *MS12*, *MS16* and *MS20* [50,51]. These markers are included in a consensus panel selected by partners within the Asia Pacific Malaria Elimination Network (www.apmen.org). The *Pv3*.*27*, *MS16* and *msp1F3* loci were amplified using methods described elsewhere [15]. The protocol for the remaining loci and the details of the primer sequences and chromosomal locations for each marker have been provided previously [15,20].

The labelled PCR products were sized by denaturing capillary electrophoresis on an ABI 3100 Genetic Analyzer with GeneScan LIZ-600 (Applied Biosystems) internal size standards. Genotype calling was facilitated with GeneMapper Version 4.0. To reduce potential artefacts, an arbitrary fluorescent intensity threshold of 50 rfu was applied for peak detection. All electropherogram traces were additionally inspected manually. For each isolate, at each locus, the predominant allele and any additional alleles with minimum 33% height of the predominant allele were scored [49].

### Population Genetic Analysis

An infection was defined as polyclonal if more than one allele was observed at one or more loci. The Multiplicity of infection (MOI) for a given sample was defined as the maximum number of alleles observed at any of the loci investigated. With the exception of measures of polyclonality and MOI, only the predominant allele at each locus in each isolate was used for analysis [49]. The expected heterozygosity (*H*_E_) was measured as an index of population diversity using the formula *H*_E_ = [*n*/ (*n*-1)] [1-Σ*p*_*i*_^2^], where *n* is the number of isolates analyzed and *pi* is the frequency of the *ith* allele in the population. The pairwise *F*_ST_ metric was used to gauge the genetic distance between populations. Calculations were undertaken using Arlequin software (version 3.5) [52]. Standardized measures (*F*’_ST_) were additionally calculated to adjust for high marker diversity [53]. Population structure was further assessed using STRUCTURE software version 2.3.3 [54]. Twenty replicates, with 100,000 burn-in and 100,000 post burn-in iterations were run for each of *K* (populations) from 1–10 using the model parameters of admixture with correlated allele frequencies. The most probable *K* was derived by applying the delta K method [55]. STRUCTURE results were displayed using bar plots prepared with *distruct* software version 1.1 [56]. Multi-locus haplotypes were reconstructed from the predominant allele at each locus in isolates with no missing data. Multi-locus linkage disequilibrium (LD) was measured by the standardised index of association (*I*_A_^S^) using the web-based LIAN 3.5 software [57]. The significance of the *I*_A_^S^ estimates was assessed using 10,000 random permutations of the data. For each population, LD was assessed in 1) all samples, 2) samples with a maximum of one multi-allelic locus, and 3) with each unique haplotype represented just once. The genetic relatedness between sample pairs was assessed by measuring the proportion of alleles shared between multi-locus haplotype pairs (*ps*). Using (1-*ps*) as a measure of genetic distance [58], an unrooted neighbour-joining tree [59] was generated with the ape package in R [60]. Mantel’s r-test was used to assess the correlation between genetic and temporal distance using the ade4 package in R [61].

### Statistical Tests

Statistical comparisons of patient gender ratio and infection polyclonality between sites and species were undertaken using Pearson’s Chi-square test. The significance of difference between sites and species in patient age, parasite density and expected heterozygosity were assessed using the Mann-Whitney U test. Statistical comparison of the MOI between sites and species was undertaken using the Kruskal-Wallis test and Mann-Whitney U test, respectively. All tests were performed using R software, with a significance threshold of 0.05.

### Ethical Approval

Ethical approval for this study was obtained from the Eijkman Institute Research Ethics Commission (EIREC) number 45/2011. Written informed consent was obtained from all adults or a parent or guardian for participants less than 18 years of age.

## Results

### Patient Sampling

Between October 2011 and July 2013, a total of 334 blood samples were collected from malaria-infected individuals across the 4 sites (Table 1). With the exception of Kalimantan, where caution is advised in data interpretation, a minimum of 30 isolates were available for each species in each site. In Bangka and Sumba, malaria-positive individuals detected by both active (ACD) and passive case detection (PCD) contributed a moderate proportion of all samples. Comparison between these sample subsets did not however identify significant differences in sample diversity for either *P*. *falciparum* or *P*. *vivax* (S2 Table). Studies in other endemic regions have found similar results [62–66]. Therefore, ACD and PCD infections were pooled for subsequent analyses.

In Bangka and Kalimantan, the majority of patients were young adults. No significant difference was observed in the median age between *P*. *vivax* (25 years, range: 1–50) and *P*. *falciparum* patients (26 years, range: 1–72) (*P* = 0.223). In Bangka, the median age of PCD patients with *P*. *vivax* (27 years, range: 3–50) was 2 years older than the ACD patients (25 years, range: 1–45) (*P* = 0.021). In the same population, a 5 year difference was observed in the median age of the *P*. *falciparum* PCD (25 years, range: 5–72) versus ACD (30 years, range: 3–50) patients but the difference was not significant (*P* = 0.389). In Sumba and West Timor, the patient demographic was younger, with a significant difference in age between *P*. *vivax* (median 6 years, range: 0–54) and *P*. *falciparum* cases (median 13 years, range: 1–67) (*P* = 3.08 x 10^–5^). In Sumba, the median age of PCD patients with *P*. *vivax* (30 years, range: 5–54) was 25 years older than the ACD patients (5 years, range: 0–30) (*P* = 3.7 x 10^–4^), although caution is advised in interpretation owing to the modest sample size of the former group (*n* = 9). The median age of *P*. *falciparum* PCD patients (15 years, range: 1–54) was also higher than the ACD patients (9 years, range: 3–65) but the difference was not significant (*P* = 0.060).

Relative to the two western provinces (78% males), the representation of male patients was less skewed in Sumba and West Timor (55% males) (*P* = 1.78 x 10^–5^). In Bangka, the proportion of males was significantly higher amongst PCD versus ACD groups in both *P*. *vivax* (92% vs 65%, *P* = 0.005) and *P*. *falciparum* (89% vs 59%, *P* = 0.021). In Sumba, no significant difference was observed in gender proportions between PCD and ACD groups in either species (both *P* >0.05).

No significant difference was observed in the median parasite density (parasites ul^-1^ blood) between *P*. *falciparum* (3,099 ul^-1^, range: 32–755,400) and *P*. *vivax* (2,110 ul^-1^, range: 48–529,600) (*P* = 0.057). In both species, parasite density was significantly lower in ACD versus PCD patients. In *P*. *vivax*, the median parasite density was 1,126 ul^-1^ (range: 48–87,770) in ACD versus 6,551 ul^-1^ (range: 73–529,600) in PCD (*P* = 1.25 x 10^–5^). In *P*. *falciparum*, the median parasite density was 1,412 ul^-1^ (range: 32–446,439) in ACD versus 21,160 ul^-1^ (range: 198–755,400) in PCD (*P* = 7.28 x 10^–11^). In *P*. *vivax*, no significant difference was observed between the sites in the parasite density in the PCD (*P* = 0.097) or ACD (*P* = 0.261) patients. In *P*. *falciparum*, no significant difference was observed between the sites in the parasite density in the PCD (*P* = 0.084) patients. A significant difference was observed amongst the *P*. *falciparum* ACD patients (*P* = 8.01 x 10^–5^), largely reflecting the low parasite density in West Timor (median = 480 ul^-1^). The various differences observed in parasite density justified the implementation of the 33% predominant peak height threshold for calling additional alleles to reduce potential bias in characterising polyclonal infections.

### Marker Properties

A summary of the genotype calls for each of the *P*. *falciparum* and *P*. *vivax* isolates in presented in the Supplementary material (S3 and S4 Tables, respectively). In *P*. *falciparum*, 12 (7.2%) and 3 (1.8%) isolates exhibited 1 and 3 genotyping failures, respectively. Whilst the TA109 and TA42 loci exhibited 9 (5.4%) and 8 (4.8%) genotyping failures, respectively, all other loci exhibited 3 or less (0–2%) failures. In *P*. *vivax*, 21 (12.5%) and 2 (1.2%) isolates exhibited 1 and 2 genotyping failures, respectively. With the exception of the *MS5* locus, which exhibited 12 (7%) genotyping failures, all other markers exhibited 3 or less (0–1%) failures.

The distribution of allele frequencies at each marker is illustrated in S1 Fig, and measures of diversity in each population are presented in S5 Table. With the exception of the TA109 locus, where the predominant allele accounted for 96.2% of all alleles, all loci exhibited a minimum of one allele with minor allele frequency ≥5%. Owing to the low level of polymorphism, the TA109 locus was excluded from further analysis. After exclusion of the TA109 locus, the only observations of monomorphic loci within populations were at the TA42 and TA60 loci in Kalimantan, and the TA1 locus in West Timor (S5 Table). These loci were therefore excluded from the analysis of LD in the given populations.

### Within-host and Population Diversity

A summary of polyclonality, MOI and population diversity is presented in Table 2. Variation was observed in the proportion of polyclonal *P*. *falciparum* infections (0–20%) and mean MOI (1–1.23) across sites, although none of the differences reached statistical significance. In contrast, the variation in the polyclonality of *P*. *vivax* infections (23–79%) was highly significant (*P* = 0.0002), as was the variation in mean MOI (1.23–1.90); *P* = 0.0002. Across the sites, higher proportions of polyclonal infections (*P* = 1.042 x 10^–12^) and higher mean MOI (*P* = 4.39 x 10^–13^) were observed in *P*. *vivax* versus *P*. *falciparum*. In *P*. *falciparum*, 41.7% (10/24) of polyclonal infections were multi-allelic at just one of the nine loci. A similar proportion of the polyclonal *P*. *vivax* infections, 38% (33/87) were multi-allelic at just one locus. In both *P*. *falciparum* and *P*. *vivax* 95% or more polyclonal infections could be identified with the seven most diverse markers.

**Table 2:** Within-host and population diversity.

Island	Species	Polyclonal Infections, %	MOI, mean (range)	*H*_E_, mean ± SE (range)
Bangka	*P*. *falciparum*	14% (8/58)	1.14 (1–2)	0.457 ± 0.072 (0.165–0.736)
	*P*. *vivax*	41% (35/86)	1.47 (1–3)	0.845 ± 0.037 (0.596–0.945)
Kalimantan	*P*. *falciparum*	0% (0/13)	1.00 (1–1)	0.397 ± 0.098 (0.000–0.731)
	*P*. *vivax*	23% (3/13)	1.23 (1–2)	0.851 ± 0.043 (0.621–0.982)
Sumba	*P*. *falciparum*	20% (12/60)	1.23 (1–3)	0.715 ± 0.069 (0.353–0.893)
	*P*. *vivax*	65% (26/40)	1.75 (1–3)	0.856 ± 0.036 (0.596–0.945)
West Timor	*P*. *falciparum*	11% (4/35)	1.11 (1–2)	0.522 ± 0.082 (0.000–0.686)
	*P*. *vivax*	79% (23/29)	1.90 (1–3)	0.806 ± 0.027 (0.670–0.916)

*P*. *falciparum* population diversity varied between sites (*H*_E_ = 0.397–0.715), with the highest diversity observed in Sumba. The differences between Sumba and each of Bangka (*P* = 0.031) and Kalimantan (*P* = 0.024) reached significance. Less variation was observed between the sites for *P*. *vivax* infections (*H*_E_ = 0.806–0.856) (all *P* > 0.05). The diversity in *P*. *vivax* was consistently higher than in sympatric *P*. *falciparum* populations, reaching significance in Bangka, Kalimantan and West Timor (all *P* ≤ 0.001) but not Sumba (*P* > 0.05).

### Population Structure and Differentiation

With the exception of Sumba and West Timor (*F*_ST_ = 0.087; *P* <1 x10^-5^), the differentiation between the *P*. *falciparum* populations was high (*F*_ST_ range: 0.238–0.415; all *P* <1 x10^-5^) (Table 3). Differentiation was lower in *P*. *vivax* (*F*_ST_ range: -0.003–0.066), with significance in all comparisons (all *P* <1 x10^-5^) with the exception of Kalimantan, possibly reflecting the limited sample size.

**Table 3:** Pair-wise differentiation between sites.

*P*. *falciparum*	Bangka	Kalimantan	Sumba	West Timor
**Bangka**	-	0.420	0.637	0.686
**Kalimantan**	0.238 (*P* <1 x10^-5^)	-	0.641	0.786
**Sumba**	0.264 (*P* <1 x10^-5^)	0.255 (*P* <1 x10^-5^)	-	0.234
**West Timor**	0.357 (*P* <1 x10^-5^)	0.415 (*P* <1 x10^-5^)	0.087 (*P* <1 x10^-5^)	-
***P*. *vivax***	**Bangka**	**Kalimantan**	**Sumba**	**West Timor**
**Bangka**	-	-0.019	0.263	0.381
**Kalimantan**	-0.003 (*P* = 0.505)	-	0.101	0.344
**Sumba**	0.039 (*P* <1 x10^-5^)	0.014 (*P* = 0.243)	-	0.362
**West Timor**	0.066 (*P* <1 x10^-5^)	0.056 (*P* = 0.036)	0.060 (*P* <1 x10^-5^)	-

***F***_**ST**_ (*P-value*) in lower left triangle. ***F*’**_**ST**_ in upper right triangle.

**Fig 2:**
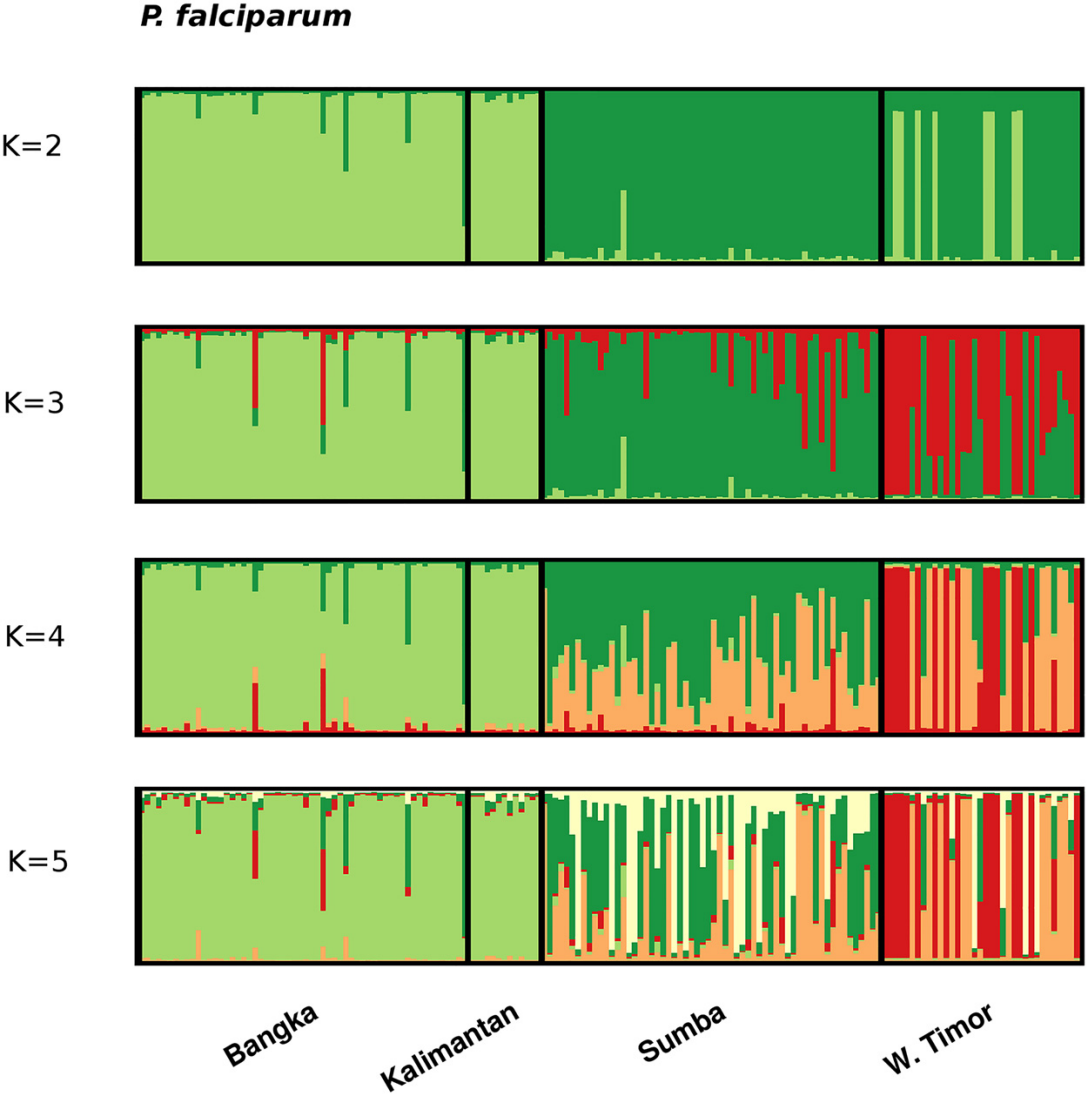
Population structure in *P falciparum*. Bar plots illustrating the population structure at *K* = 2–5 in *P*. *falciparum*. Each vertical bar represents an individual sample and each colour represents one of the *K* clusters (sub-populations) defined by STRUCTURE. For each sample, the predicted ancestry to each of the K sub-populations is represented by the colour-coded bars. *K*1 = light green, *K*2 = dark green, *K*3 = red, *K*4 = orange, and *K*5 = white.

STRUCTURE analysis demonstrated clear sub-structure in the *P*. *falciparum* population (Fig 2). The delta K method demonstrated *K* = 2 as having the greatest likelihood (S2 Fig). At *K* = 2, the large majority of isolates from Bangka and Kalimantan, and approximately one third from West Timor demonstrated predominant ancestry to the *K*1 cluster, whilst all isolates from Sumba and the remaining two thirds from West Timor demonstrated predominant ancestry to the *K*2 cluster. At higher estimates of *K*, greater distinction was observed between the two eastern (Sumba and West Timor) populations, and increasing sub-structure was observed within West Timor.

**Fig 3:**
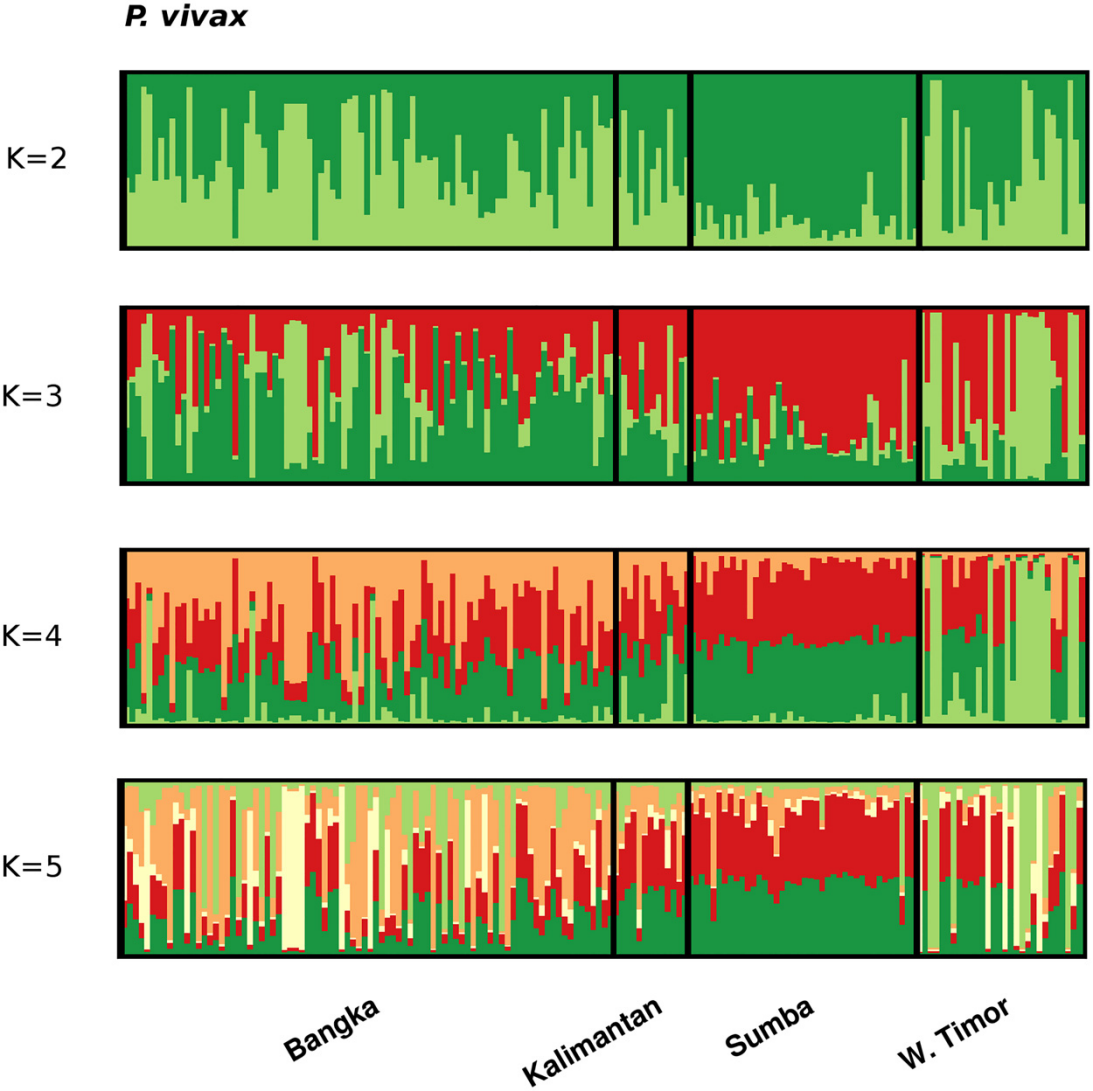
Population structure in *P*. *vivax*. Bar plots illustrating the population structure at *K* = 2–5 in *P*. *vivax*. *K*1 = light green, *K*2 = dark green, *K*3 = red, *K*4 = orange, and *K*5 = white.

In *P*. *vivax*, the delta K method identified *K* = 5 as the most likely number of sub-populations (S2 Fig). However, the large majority of isolates from all 4 populations demonstrated mixed ancestry to the various clusters (Fig 3).

### Relatedness

**Fig 4:**
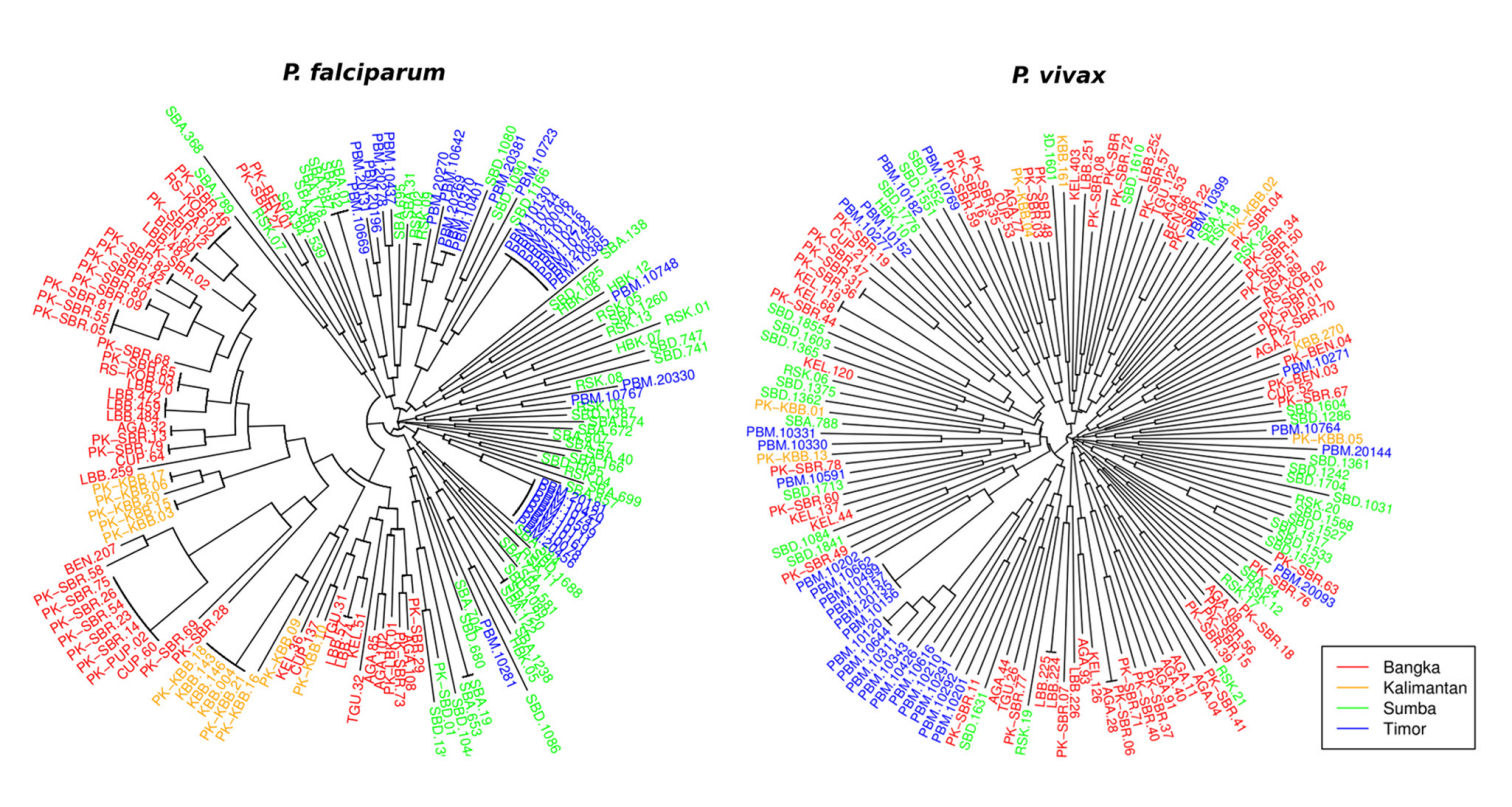
Unrooted neighbour-joining tree illustrating the genetic relatedness between *P*. *falciparum* (top) and *P*. *vivax* (bottom) isolates.

Neighbour-joining analysis demonstrated moderately distinct separation between the western and eastern *P*. *falciparum* isolates (Fig 4). Clusters of isolates with 4 or more identical or near-identical multi-locus haplotypes were observed in Bangka and West Timor, whilst the Sumba isolates were generally less related to one another.

Fewer identical isolates were observed in *P*. *vivax* relative to *P*. *falciparum*. The isolates from West Timor largely clustered together, and four identical isolates were observed in this population. Two of the identical isolates were collected on the same day, and the other two were both collected four days later. Within these sampling dates, other more distinct isolates were also collected. Nonetheless, a significant correlation was observed between the distance in sampling date and the proportion of alleles shared between the *P*. *vivax* infections in West Timor (Mantel r-test, *r* = 0.12, *P* = 0.02). Relative to *P*. *falciparum*, little distinction was observed between the *P*. *vivax* infections from the other three sites. With the exception of the *P*. *vivax* population in West Timor, no significant correlation was observed between the distance in sampling date and the proportion of alleles shared between infections for *P*. *falciparum* or *P*. *vivax*.

### Linkage Disequilibrium

In *P*. *falciparum*, LD ranged from an *I*_A_^S^ of 0.018 to 0.239 (all *P* ≤ 0.05) (Table 4). However, after removing duplicate haplotypes, the *I*_A_^S^ levels dropped more than two-fold in each of Bangka, Kalimantan, and West Timor.

**Table 4:** Linkage disequilibrium.

		All infections		Low complexity ^1^		Unique haplotypes	
Island	Species	*N*	*I*_A_^S^	*N*	*I*_A_^S^	*N*	*I*_A_^S^
Bangka	*P*. *falciparum*	53	0.048**	47 (88%)	0.062**	32 (60%)	0.010 ^NS^
	*P*. *vivax*	73	0.032**	51 (70%)	0.034**	69 (95%)	0.023**
Kalimantan	*P*. *falciparum*	13	0.239 ***	13 (100%)	0.239 ***	7 (54%)	0.001 ^NS^
	*P*. *vivax*	7	-0.003 ^NS^	6 (86%)	-0.016 ^NS^	7 (100%)	-0.003 ^NS^
Sumba	*P*. *falciparum*	59	0.018*	53 (91%)	0.025 *	54 (93%)	0.011 ^NS^
	*P*. *vivax*	37	0.017*	20 (54%)	0.039 *	37 (100%)	0.017 *
West Timor	*P*. *falciparum*	32	0.229**	31 (96.9%)	0.226**	16 (50%)	0.094**
	*P*. *vivax*	28	0.188**	20 (71%)	0.263**	24 (86%)	0.089 **

Only samples with no missing data are included in the analyses. All nine loci were used in all *P*. *vivax* populations. In the *P*. *falciparum* populations, 8 loci were analysed in Bangka and Sumba, 6 in Kalimantan (exclusion of monomorphic loci TA42 and TA60), and 7 loci in West Timor (exclusion of monomorphic loci TA1).

^1^ Restricted multi-locus haplotypes from samples with no more than one multi-allelic locus.

^NS^ Not significant (*P* > 0.05)

* *P* < 0.05

** *P* < 0.01

*** *P* < 0.001.

In *P*. *vivax*, significant LD was observed in Bangka, Sumba and West Timor (*I*_A_^S^ = 0.017–0.188; all *P* < = 0.003), but not Kalimantan (*I*_A_^S^ = -0.003; *P* > 0.05). Identical isolates were less frequent than in *P*. *falciparum*, and the *I*_A_^S^ scores from the unique multi-locus data sets retained equivalent significance to the unfiltered data sets.

## Discussion

The heterogeneous epidemiology of malaria in Indonesia provided an opportunity to investigate the comparative transmission dynamics of co-endemic *P*. *vivax* and *P*. *falciparum* isolates in multiple settings. Using molecular approaches, we demonstrated striking contrasts in diversity and population structure, indicative of varying transmission patterns within and between the two species in different endemicities.

A range of patterns were observed in *P*. *falciparum* diversity and structure across the sites. In all populations, polyclonal infection rates (0–20%) were comparable to rates observed in low endemic settings in Southeast Asia [17,23], South America [16,26] and post-intervention sites elsewhere [19,30], suggestive of low transmission and infrequent superinfection. Greater variation was observed in the population diversity (*H*_E_ = 0.397–0.715). The highest level of diversity was observed in Sumba, reflecting the higher prevalence in this population relative to the other three sites. Whilst the population diversity in Sumba was comparable to levels observed in hyper-holoendemic regions common to Africa [16,27,30], Bangka, Kalimantan and West Timor were more similar to hypo-mesoendemic and unstable endemic regions of South America [16,26] and Southeast Asia [17,23,29]. Relative to Sumba, the latter three populations also exhibited higher levels of LD.

Owing to pooling of *P*. *falciparum* samples collected from two regencies (West and Central Bangka), as well as population sub-structure, one possible explanation for the significant LD observed in Bangka was admixture. However, STRUCTURE analysis demonstrated that the majority of isolates in Bangka exhibited predominant ancestry to a single population. In contrast, although sampled from a single administrative district, STRUCTURE analysis indicated that the West Timor population comprised isolates from at least two sub-populations. Thus, admixture may have contributed to the LD observed in West Timor. As demonstrated by the greater than two-fold decline in the index of association (*I*_A_^S^) after accounting for identical multi-locus haplotypes, epidemic expansions also appear to have contributed to the LD observed in West Timor, as well as in Bangka, and Kalimantan. In accordance with epidemic dynamics, clusters of isolates with identical multi-locus haplotypes were prevalent in these populations. Whilst it is possible that the modest number of loci reduced the ability to discriminate between different ‘strains‘, the high diversity observed in Sumba suggests that the resolving capacity was adequate.

In concert, the observed trends in diversity and LD demonstrated a pattern of low, epidemic *P*. *falciparum* transmission in Bangka, Kalimantan and West Timor. These trends are consistent with Ministry of Health data on malaria incidence in these regions, where *P*. *falciparum* endemicity is characterised as unstable endemic to hypo-mesoendemic. Relative to Sumba, patients from Bangka, Kalimantan and West Timor were older (largely teenagers and young adults), with a higher proportion of males in Bangka and Kalimantan. These demographics might in part reflect at-risk populations such as particular occupational groups, and might explain some of the epidemic transmission dynamics. In contrast to the other populations, the high diversity and linkage equilibrium in Sumba indicated that local *P*. *falciparum* transmission remained disconcertingly high and stable in this region. In accordance, the endemicity in Sumba lay at the higher end of the hypo-mesoendemic spectrum, and the *P*. *falciparum* patient demographic largely reflected children, with limited gender bias.

As discussed with the specific examples from our data in Indonesia, the heterogeneity in the dynamics of *P*. *falciparum* transmission across populations has important implications for elimination. Although Sumba and West Timor are grouped in the same target category for elimination by the national malaria elimination program (elimination by 2020), our data demonstrates that they would likely benefit from different intervention activities. The *P*. *falciparum* dynamics in Sumba demonstrate the need for total coverage intervention activities such as broad-ranging distribution of long lasting insecticide-treated bed nets. In contrast, more targeted approaches such as reactive case detection and treatment or indoor residual spraying targeted to households in transmission hot-spots or at-risk populations would be more cost-effective in West Timor [14]. We note that present MOH policies in these regions, based upon passively reported confirmed malaria cases, are consistent with the results of our genetic analysis. With greater sample size, further investigation of both the parasite and patient dynamics should enable identification of at-risk populations for target intervention in West Timor, Bangka and Kalimantan.

Differentiation between the *P*. *falciparum* populations was generally high, comparable to levels observed in Malaysian Borneo, where declining transmission and limited migration led to a fragmented parasite population [17]. The declining transmission, distance and island setting should all have facilitated the isolation of the parasite populations assessed here. However, differentiation remained moderate between Sumba and West Timor, highlighting the challenge of parasite introductions into West Timor from the stable transmission setting of Sumba. Indeed, the demonstration of multiple distinct sub-populations by STRUCTURE analysis indicated that other populations such as neighbouring Timor-Leste may present additional reservoirs of infection to West Timor. Identifying the major reservoirs sustaining infection in such populations will be paramount to the success of Indonesia’s elimination campaign. Other global populations will no doubt face similar challenges in the strive toward elimination.

Albeit less marked than in *P*. *falciparum*, variation was also observed between sites in *P*. *vivax* diversity and structure. Polyclonal infection rates ranged from 23 to 79%, in broad agreement with local API estimates, indicating potential utility in informing on transmission intensity. In the eastern provinces, where patient demographics largely reflected young children, within-host diversity was comparable to high transmission regions of the Pacific [24], Southeast Asia [20,28] and the Horn of Africa [20]. In the western provinces, patient demographics were skewed toward young adults, with a high proportion of males in Bangka. In these populations, within-host diversity was more comparable to post-intervention sites in the Pacific [19] and Malaysia [15]. Relative to the sympatric *P*. *falciparum* populations, within-host diversity was consistently higher in *P*. *vivax*, in accordance with other studies [18,19,28]. The available evidence suggests that the hypnozoite reservoir and the early, often pre-treatment, development of gametocytes in *P*. *vivax* likely explain these observations, and thus have implications for *P*. *vivax* control and elimination in other endemic populations.

In contrast to within-host diversity, as demonstrated in other studies across a range of endemic sites across the globe [18–22,24,25,33,50], *P*. *vivax* population diversity was high in all four sites (*H*_E_ = 0.81–0.86), with no apparent correlation with endemicity. Furthermore, the *P*. *vivax* populations demonstrated consistently higher diversity than their sympatric *P*. *falciparum* populations. The factor(s) responsible for these trends remain unclear. A plausible explanation is that relapsing infections, by enhancing infection complexity, increase the probability that different clones are taken up in the mosquito blood meal, thus promoting the opportunity for new variants to be generated via recombination. However, in temperate regions such as Central China and South Korea, relapse rates are low (20%) and yet *P*. *vivax* population diversity remains comparably high [22,25]. Another non-mutually exclusive possibility is that imported infections sustain the diversity of local *P*. *vivax* populations [22,67].

The observation of significant LD in *P*. *vivax* populations with extensive within-host and population diversity presents a conundrum also encountered in other studies [18,20,21,28,34,67]. Possible explanations for this include a frequently clonal (non-recombining) mode of propagation and/or potential artefact such as selection at or near several of the markers used [28].

In contrast to the *P*. *falciparum* populations, the LD patterns in *P*. *vivax* did not demonstrate evidence of epidemic transmission in any of the populations investigated. In concert with the observed differences in within-host and population diversity, the differences in LD demonstrated that in several of the endemic settings investigated here, the *P*. *vivax* isolates exhibited more frequent and stable transmission than their *P*. *falciparum* counterparts. Hence different interventions may be optimal for the respective malaria species in certain settings; local intervention strategies will need to reconcile these differences to ensure adequate impact against all species of malaria. For example, in Bangka, it appears that ongoing interventions have effectively reduced the intensity of local *P*. *falciparum* transmission. The current dynamics are moderately unstable with frequent clonal outbreaks amenable to containment by targeted approaches such as reactive case detection and treatment, and indoor residual spraying of case and surrounding households where the predominant vector species behaviour is compatible, prioritizing cases with rapidly emerging outbreak strains. However, the available evidence indicates that targeted approaches would be less effective for the co-endemic *P*. *vivax* population. Likely owing to biological differences between the two species such as the hypnozoite stage or comparatively early development of gametocytes in *P*. *vivax* versus *P*. *falciparum*, the Bangka *P*. *vivax* population appeared to have been less impacted by ongoing interventions than the *P*. *falciparum* population, displaying more stable transmission with no evidence of clonal outbreaks. These patterns suggest that broad ranging interventions such as large-scale long lasting insecticide-treated bed net coverage are still needed against *P*. *vivax*. In addition, effective treatment of the dormant liver stage may be critical to the success of *P*. *vivax* intervention efforts.

In contrast to *P*. *falciparum*, genetic differentiation was generally low in *P*. *vivax*. This observation might reflect the enhanced ‘mobility’ of the parasite afforded by the asymptomatic hypnozoite reservoir, with important implications for the containment of infection, particularly with the rise of anti-malarial drug resistance in *P*. *vivax* infection [68]. Alternatively, as discussed by Koepfli and Orjuela-Sanchez, observed trends in *P*. *vivax* population diversity and differentiation may in part reflect older, historical events [24,28]. A better understanding of the mechanisms sustaining the diversity, and limiting the observed genetic differentiation between geographically isolated *P*. *vivax* populations will be critical to the successful elimination of this species.

### Conclusions

Concerted efforts are required to target the major reservoirs of *P*. *vivax* infection if malaria elimination is to be successful in co-endemic regions. A better understanding of the local variation in how frequently parasites are transmitted, how focal these transmission events are in time and space, and the ‘mobility’, or potential for parasite spread, can facilitate more optimal malaria intervention strategies. Parasite genotyping can provide useful insights on these dynamics, providing a complementary surveillance tool to the more traditional epidemiological measures, particularly in regions where non-falciparum species circulate.

## Supporting Information

S1 Table Site details. ^1^ 2010 Census.^2^ Annual parasite incidence (API) expressed as cases per 1000 population of the administrative region examined (year of investigation and percentage of the population at risk examined are provided in parentheses). Details on the API were provided by Dr Iqbal Elyazar, Malaria Atlas Project.
(DOCX)

S2 Table Within-host and population diversity in *P*. *falciparum* and *P*. *vivax* in passive versus actively detected cases from Bangka and Sumba. (DOCX)

S3 Table Genotype calls at 9 markers in 166 *P*. *falciparum* isolates. (CSV)

S4 Table Genotype calls at 9 markers in 168 *P*. *vivax* isolates. (CSV)

S5 Table Marker diversity in *P*. *falciparum* and *P*. *vivax*. (DOCX)

S1 Fig Allele frequency distributions at 9 markers in 157–166 *P*. *falciparum*, and 9 markers in 156–168 *P*. *vivax* samples. (TIF)

S2 Fig Delta K method: *ΔK* against *K*. (TIFF)

## References

[1] Guerra CA, Howes RE, Patil AP, Gething PW, Van Boeckel TP, et al (2010) The international limits and population at risk of Plasmodium vivax transmission in 2009. PLoS Negl Trop Dis 4: e774 10.1371/journal.pntd.0000774 20689816 PMC2914753

[2] Baird JK (2009) Severe and fatal vivax malaria challenges 'benign tertian malaria' dogma. Ann Trop Paediatr 29: 251–252. 10.1179/027249309X12547917868808 19941746

[3] Baird JK (2009) Resistance to therapies for infection by Plasmodium vivax. Clin Microbiol Rev 22: 508–534. 10.1128/CMR.00008-09 19597012 PMC2708388

[4] Carlton JM, Sina BJ, Adams JH (2011) Why is Plasmodium vivax a neglected tropical disease? PLoS Negl Trop Dis 5: e1160 10.1371/journal.pntd.0001160 21738804 PMC3125139

[5] Mueller I, Galinski MR, Baird JK, Carlton JM, Kochar DK, et al (2009) Key gaps in the knowledge of Plasmodium vivax, a neglected human malaria parasite. Lancet Infect Dis 9: 555–566. 10.1016/S1473-3099(09)70177-X 19695492

[6] Price RN, von Seidlein L, Valecha N, Nosten F, Baird JK, et al (2014) Global extent of chloroquine-resistant Plasmodium vivax: a systematic review and meta-analysis. Lancet Infect Dis 14: 982–991. 10.1016/S1473-3099(14)70855-2 25213732 PMC4178238

[7] Tjitra E, Anstey NM, Sugiarto P, Warikar N, Kenangalem E, et al (2008) Multidrug-resistant Plasmodium vivax associated with severe and fatal malaria: a prospective study in Papua, Indonesia. PLoS Med 5: e128 10.1371/journal.pmed.0050128 18563962 PMC2429950

[8] Menard D, Barnadas C, Bouchier C, Henry-Halldin C, Gray LR, et al (2010) Plasmodium vivax clinical malaria is commonly observed in Duffy-negative Malagasy people. Proc Natl Acad Sci U S A 107: 5967–5971. 10.1073/pnas.0912496107 20231434 PMC2851935

[9] Mendes C, Dias F, Figueiredo J, Mora VG, Cano J, et al (2011) Duffy negative antigen is no longer a barrier to Plasmodium vivax—molecular evidences from the African West Coast (Angola and Equatorial Guinea). PLoS Negl Trop Dis 5: e1192 10.1371/journal.pntd.0001192 21713024 PMC3119644

[10] Ryan JR, Stoute JA, Amon J, Dunton RF, Mtalib R, et al (2006) Evidence for transmission of Plasmodium vivax among a duffy antigen negative population in Western Kenya. Am J Trop Med Hyg 75: 575–581. 17038676

[11] Woldearegai TG, Kremsner PG, Kun JF, Mordmuller B (2013) Plasmodium vivax malaria in Duffy-negative individuals from Ethiopia. Trans R Soc Trop Med Hyg 107: 328–331. 10.1093/trstmh/trt016 23584375

[12] Cavasini CE, de Mattos LC, Couto AA, Couto VS, Gollino Y, et al (2007) Duffy blood group gene polymorphisms among malaria vivax patients in four areas of the Brazilian Amazon region. Malar J 6: 167 18093292 10.1186/1475-2875-6-167 PMC2244634

[13] Feachem RG, Phillips AA, Hwang J, Cotter C, Wielgosz B, et al (2010) Shrinking the malaria map: progress and prospects. Lancet 376: 1566–1578. 10.1016/S0140-6736(10)61270-6 21035842 PMC3044848

[14] Hay SI, Smith DL, Snow RW (2008) Measuring malaria endemicity from intense to interrupted transmission. Lancet Infect Dis 8: 369–378. 10.1016/S1473-3099(08)70069-0 18387849 PMC2653619

[15] Abdullah NR, Barber BE, William T, Norahmad NA, Satsu UR, et al (2013) Plasmodium vivax population structure and transmission dynamics in Sabah Malaysia. PLoS One 8: e82553 10.1371/journal.pone.0082553 24358203 PMC3866266

[16] Anderson TJ, Haubold B, Williams JT, Estrada-Franco JG, Richardson L, et al (2000) Microsatellite markers reveal a spectrum of population structures in the malaria parasite Plasmodium falciparum. Mol Biol Evol 17: 1467–1482. 11018154 10.1093/oxfordjournals.molbev.a026247

[17] Anthony TG, Conway DJ, Cox-Singh J, Matusop A, Ratnam S, et al (2005) Fragmented population structure of plasmodium falciparum in a region of declining endemicity. J Infect Dis 191: 1558–1564. 15809916 10.1086/429338

[18] Ferreira MU, Karunaweera ND, da Silva-Nunes M, da Silva NS, Wirth DF, et al (2007) Population structure and transmission dynamics of Plasmodium vivax in rural Amazonia. J Infect Dis 195: 1218–1226. 17357061 10.1086/512685

[19] Gray KA, Dowd S, Bain L, Bobogare A, Wini L, et al (2013) Population genetics of Plasmodium falciparum and Plasmodium vivax and asymptomatic malaria in Temotu Province, Solomon Islands. Malar J 12: 429 10.1186/1475-2875-12-429 24261646 PMC4222835

[20] Gunawardena S, Karunaweera ND, Ferreira MU, Phone-Kyaw M, Pollack RJ, et al (2010) Geographic structure of Plasmodium vivax: microsatellite analysis of parasite populations from Sri Lanka, Myanmar, and Ethiopia. Am J Trop Med Hyg 82: 235–242. 10.4269/ajtmh.2010.09-0588 20133999 PMC2813164

[21] Imwong M, Nair S, Pukrittayakamee S, Sudimack D, Williams JT, et al (2007) Contrasting genetic structure in Plasmodium vivax populations from Asia and South America. Int J Parasitol 37: 1013–1022. 17442318 10.1016/j.ijpara.2007.02.010

[22] Iwagami M, Fukumoto M, Hwang SY, Kim SH, Kho WG, et al (2012) Population structure and transmission dynamics of Plasmodium vivax in the Republic of Korea based on microsatellite DNA analysis. PLoS Negl Trop Dis 6: e1592 10.1371/journal.pntd.0001592 22509416 PMC3317904

[23] Iwagami M, Rivera PT, Villacorte EA, Escueta AD, Hatabu T, et al (2009) Genetic diversity and population structure of Plasmodium falciparum in the Philippines. Malar J 8: 96 10.1186/1475-2875-8-96 19422722 PMC2685811

[24] Koepfli C, Timinao L, Antao T, Barry AE, Siba P, et al (2013) A Large Reservoir and Little Population Structure in the South Pacific. PLoS One 8: e66041 23823758 10.1371/journal.pone.0066041 PMC3688846

[25] Liu Y, Auburn S, Cao J, Trimarsanto H, Zhou H, et al (2014) Genetic diversity and population structure of Plasmodium vivax in Central China. Malar J 13: 262 10.1186/1475-2875-13-262 25008859 PMC4094906

[26] Machado RL, Povoa MM, Calvosa VS, Ferreira MU, Rossit AR, et al (2004) Genetic structure of Plasmodium falciparum populations in the Brazilian Amazon region. J Infect Dis 190: 1547–1555. 15478058 10.1086/424601

[27] Mobegi VA, Loua KM, Ahouidi AD, Satoguina J, Nwakanma DC, et al (2012) Population genetic structure of Plasmodium falciparum across a region of diverse endemicity in West Africa. Malar J 11: 223 10.1186/1475-2875-11-223 22759447 PMC3425276

[28] Orjuela-Sanchez P, Sa JM, Brandi MC, Rodrigues PT, Bastos MS, et al (2013) Higher microsatellite diversity in Plasmodium vivax than in sympatric Plasmodium falciparum populations in Pursat, Western Cambodia. Exp Parasitol 134: 318–326. 10.1016/j.exppara.2013.03.029 23562882 PMC3691688

[29] Pumpaibool T, Arnathau C, Durand P, Kanchanakhan N, Siripoon N, et al (2009) Genetic diversity and population structure of Plasmodium falciparum in Thailand, a low transmission country. Malar J 8: 155 10.1186/1475-2875-8-155 19602241 PMC2722663

[30] Rebaudet S, Bogreau H, Silai R, Lepere JF, Bertaux L, et al (2010) Genetic structure of Plasmodium falciparum and elimination of malaria, Comoros archipelago. Emerg Infect Dis 16: 1686–1694. 10.3201/eid1611.100694 21029525 PMC3294527

[31] Rezende AM, Tarazona-Santos E, Couto AD, Fontes CJ, De Souza JM, et al (2009) Analysis of genetic variability of Plasmodium vivax isolates from different Brazilian Amazon areas using tandem repeats. Am J Trop Med Hyg 80: 729–733. 19407114

[32] Schousboe ML, Ranjitkar S, Rajakaruna RS, Amerasinghe PH, Konradsen F, et al (2014) Global and local genetic diversity at two microsatellite loci in Plasmodium vivax parasites from Asia, Africa and South America. Malar J 13: 392 10.1186/1475-2875-13-392 25277367 PMC4200131

[33] Van den Eede P, Erhart A, Van der Auwera G, Van Overmeir C, Thang ND, et al (2010) High complexity of Plasmodium vivax infections in symptomatic patients from a rural community in central Vietnam detected by microsatellite genotyping. Am J Trop Med Hyg 82: 223–227. 10.4269/ajtmh.2010.09-0458 20133996 PMC2813161

[34] Van den Eede P, Van der Auwera G, Delgado C, Huyse T, Soto-Calle VE, et al (2010) Multilocus genotyping reveals high heterogeneity and strong local population structure of the Plasmodium vivax population in the Peruvian Amazon. Malar J 9: 151 10.1186/1475-2875-9-151 20525233 PMC2898784

[35] Schultz L, Wapling J, Mueller I, Ntsuke PO, Senn N, et al (2010) Multilocus haplotypes reveal variable levels of diversity and population structure of Plasmodium falciparum in Papua New Guinea, a region of intense perennial transmission. Malar J 9: 336 10.1186/1475-2875-9-336 21092231 PMC3002378

[36] Arnott A, Barry AE, Reeder JC (2012) Understanding the population genetics of Plasmodium vivax is essential for malaria control and elimination. Malar J 11: 14 10.1186/1475-2875-11-14 22233585 PMC3298510

[37] World Health Organization (2013) World Malaria Report 2013. World Health Organization; Geneva 2013.

[38] Ratcliff A, Siswantoro H, Kenangalem E, Wuwung M, Brockman A, et al (2007) Therapeutic response of multidrug-resistant Plasmodium falciparum and P. vivax to chloroquine and sulfadoxine-pyrimethamine in southern Papua, Indonesia. Trans R Soc Trop Med Hyg 101: 351–359. 17028048 10.1016/j.trstmh.2006.06.008 PMC2080856

[39] Sumawinata IW, Bernadeta, Leksana B, Sutamihardja A, Purnomo, et al (2003) Very high risk of therapeutic failure with chloroquine for uncomplicated Plasmodium falciparum and P. vivax malaria in Indonesian Papua. Am J Trop Med Hyg 68: 416–420. 12875290

[40] Elyazar IR, Hay SI, Baird JK (2011) Malaria distribution, prevalence, drug resistance and control in Indonesia. Adv Parasitol 74: 41–175. 10.1016/B978-0-12-385897-9.00002-1 21295677 PMC3075886

[41] Sulistyaningsih E, Fitri LE, Loscher T, Berens-Riha N (2010) Diagnostic difficulties with Plasmodium knowlesi infection in humans. Emerg Infect Dis 16: 1033–1034. 10.3201/eid1606.100022 20507769 PMC3086231

[42] Figtree M, Lee R, Bain L, Kennedy T, Mackertich S, et al (2010) Plasmodium knowlesi in human, Indonesian Borneo. Emerg Infect Dis 16: 672–674. 10.3201/eid1604.091624 20350383 PMC3321967

[43] Sinka ME, Bangs MJ, Manguin S, Rubio-Palis Y, Chareonviriyaphap T, et al (2012) A global map of dominant malaria vectors. Parasit Vectors 5: 69 10.1186/1756-3305-5-69 22475528 PMC3349467

[44] Malaria Atlas Project website. Available: www.map.ox.ac.uk. Accessed 23rd February, 2015.

[45] Gething PW, Patil AP, Smith DL, Guerra CA, Elyazar IR, et al (2011) A new world malaria map: Plasmodium falciparum endemicity in 2010. Malar J 10: 378 10.1186/1475-2875-10-378 22185615 PMC3274487

[46] Gething PW, Elyazar IR, Moyes CL, Smith DL, Battle KE, et al (2012) A long neglected world malaria map: Plasmodium vivax endemicity in 2010. PLoS Negl Trop Dis 6: e1814 10.1371/journal.pntd.0001814 22970336 PMC3435256

[47] Snounou G, Viriyakosol S, Jarra W, Thaithong S, Brown KN (1993) Identification of the four human malaria parasite species in field samples by the polymerase chain reaction and detection of a high prevalence of mixed infections. Mol Biochem Parasitol 58: 283–292. 8479452 10.1016/0166-6851(93)90050-8

[48] Boonma P, Christensen PR, Suwanarusk R, Price RN, Russell B, et al (2007) Comparison of three molecular methods for the detection and speciation of Plasmodium vivax and Plasmodium falciparum. Malar J 6: 124 17868467 10.1186/1475-2875-6-124 PMC2020467

[49] Anderson TJ, Su XZ, Bockarie M, Lagog M, Day KP (1999) Twelve microsatellite markers for characterization of Plasmodium falciparum from finger-prick blood samples. Parasitology 119 (Pt 2): 113–125. 10466118 10.1017/s0031182099004552

[50] Karunaweera ND, Ferreira MU, Munasinghe A, Barnwell JW, Collins WE, et al (2008) Extensive microsatellite diversity in the human malaria parasite Plasmodium vivax. Gene 410: 105–112. 10.1016/j.gene.2007.11.022 18226474

[51] Koepfli C, Mueller I, Marfurt J, Goroti M, Sie A, et al (2009) Evaluation of Plasmodium vivax genotyping markers for molecular monitoring in clinical trials. J Infect Dis 199: 1074–1080. 10.1086/597303 19275476

[52] Excoffier L, Lischer HE (2010) Arlequin suite ver 3.5: a new series of programs to perform population genetics analyses under Linux and Windows. Mol Ecol Resour 10: 564–567. 10.1111/j.1755-0998.2010.02847.x 21565059

[53] Hedrick PW (2005) A standardized genetic differentiation measure. Evolution 59: 1633–1638. 16329237

[54] Pritchard JK, Stephens M, Donnelly P (2000) Inference of population structure using multilocus genotype data. Genetics 155: 945–959. 10835412 10.1093/genetics/155.2.945 PMC1461096

[55] Evanno G, Regnaut S, Goudet J (2005) Detecting the number of clusters of individuals using the software STRUCTURE: a simulation study. Mol Ecol 14: 2611–2620. 15969739 10.1111/j.1365-294X.2005.02553.x

[56] Rosenberg NA (2004) *Distruct*: a program for the graphical display of population structure. Molecular Ecology Notes 4: 137–138.

[57] Haubold B, Hudson RR (2000) LIAN 3.0: detecting linkage disequilibrium in multilocus data. Linkage Analysis. Bioinformatics 16: 847–848. 11108709 10.1093/bioinformatics/16.9.847

[58] Bowcock AM, Ruiz-Linares A, Tomfohrde J, Minch E, Kidd JR, et al (1994) High resolution of human evolutionary trees with polymorphic microsatellites. Nature 368: 455–457. 7510853 10.1038/368455a0

[59] Saitou N, Nei M (1987) The neighbor-joining method: a new method for reconstructing phylogenetic trees. Mol Biol Evol 4: 406–425. 3447015 10.1093/oxfordjournals.molbev.a040454

[60] Paradis E, Claude J, Strimmer K (2004) APE: Analyses of Phylogenetics and Evolution in R language. Bioinformatics 20: 289–290. 14734327 10.1093/bioinformatics/btg412

[61] Dray S, Dufour AB (2007) The ade4 package: implementing the duality diagram for ecologists. Journal of Statistical Software 22: 1–20.

[62] Amodu OK, Oyedeji SI, Ntoumi F, Orimadegun AE, Gbadegesin RA, et al (2008) Complexity of the msp2 locus and the severity of childhood malaria, in south-western Nigeria. Ann Trop Med Parasitol 102: 95–102. 10.1179/136485908X252340 18318931

[63] Beck HP, Felger I, Huber W, Steiger S, Smith T, et al (1997) Analysis of multiple Plasmodium falciparum infections in Tanzanian children during the phase III trial of the malaria vaccine SPf66. J Infect Dis 175: 921–926. 9086150 10.1086/513991

[64] Kyes S, Harding R, Black G, Craig A, Peshu N, et al (1997) Limited spatial clustering of individual Plasmodium falciparum alleles in field isolates from coastal Kenya. Am J Trop Med Hyg 57: 205–215. 9288818 10.4269/ajtmh.1997.57.205

[65] Roper C, Richardson W, Elhassan IM, Giha H, Hviid L, et al (1998) Seasonal changes in the Plasmodium falciparum population in individuals and their relationship to clinical malaria: a longitudinal study in a Sudanese village. Parasitology 116 (Pt 6): 501–510. 9651932 10.1017/s0031182098002650

[66] Zwetyenga J, Rogier C, Tall A, Fontenille D, Snounou G, et al (1998) No influence of age on infection complexity and allelic distribution in Plasmodium falciparum infections in Ndiop, a Senegalese village with seasonal, mesoendemic malaria. Am J Trop Med Hyg 59: 726–735. 9840589 10.4269/ajtmh.1998.59.726

[67] Gunawardena S, Ferreira MU, Kapilananda GM, Wirth DF, Karunaweera ND (2014) The Sri Lankan paradox: high genetic diversity in Plasmodium vivax populations despite decreasing levels of malaria transmission. Parasitology 141: 880–890. 10.1017/S0031182013002278 24533989 PMC7485621

[68] Price RN, Auburn S, Marfurt J, Cheng Q (2012) Phenotypic and genotypic characterisation of drug-resistant Plasmodium vivax. Trends Parasitol 28: 522–529. 10.1016/j.pt.2012.08.005 23044287 PMC4627502

