# Supplementary material for: Contrasting Transmission Dynamics of Co-endemic Plasmodium vivax and P. falciparum: Implications for Malaria Control and Elimination
Source: PLoS Negl Trop Dis. 2015 May 7;9(5):e0003739. doi: 10.1371/journal.pntd.0003739 (PMC4423885; doi:10.1371/journal.pntd.0003739)
Supplement: S1 Table — 1 2010 Census.2 Annual parasite incidence (API) expressed as cases per 1000 population of the administrative region examined (year of investigation and percentage of the population at risk examined are provided in parentheses). Details on the API were provided by Dr Iqbal Elyazar, Malaria Atlas Project. (DOCX) [file pntd.0003739.s001.docx]

**Table S1. Site details**

| **Site Name** | **Sampling locations** | **Area, km^2^** | **Population Size ^1^** | ***Plasmodium* spp.** | **API** ^2^ |
| --- | --- | --- | --- | --- | --- |
| Bangka | West Bangka Regency | 2,821 | 175,110 | *P. falciparum* | 6.68 (2010, 8%) |
|  |  |  |  | *P. vivax* | 6.79 (2010, 8%) |
|  | Central Bangka Regency | 2,126 | 172,476 | *P. falciparum* | 3.36 (2010, 3%) |
|  |  |  |  | *P. vivax* | 2.21 (2010, 3%) |
| Kalimantan | Balai-Berkuak, Ketapang Regency | 3,175 | 27,739 | *P. falciparum* | 0.13 (2012, 4%) |
|  |  |  |  |  | 0.31 (2013, 2%) |
|  |  |  |  | *P. vivax* | 0.70 (2012, 4%) |
|  |  |  |  |  | 1.42 (2013, 2%) |
| Sumba | West Sumba Regency | 737 | 111,023 | *P. falciparum* | 13.78 (2010, 14%) |
|  |  |  |  | *P. vivax* | 32.47 (2010, 14%) |
|  | Southwest Sumba Regency | 1,445 | 283,818 | *P. falciparum* | 5.22 (2010, 7%) |
|  |  |  |  | *P. vivax* | 26.10 (2010, 7%) |
| West Timor | Belu Regency | 2,446 | 352,400 | *P. falciparum* | 9.72 (2010, 13%) |
|  |  |  |  | *P. vivax* | 13.26 (2010, 13%) |

^1^ 2010 Census.^2^ Annual parasite incidence (API) expressed as cases per 1000 population of the administrative region examined (year of investigation and percentage of the population at risk examined are provided in parentheses). Details on the API were provided by Dr Iqbal Elyazar, Malaria Atlas Project.
