# Supplementary material for: Contrasting Transmission Dynamics of Co-endemic Plasmodium vivax and P. falciparum: Implications for Malaria Control and Elimination
Source: PLoS Negl Trop Dis. 2015 May 7;9(5):e0003739. doi: 10.1371/journal.pntd.0003739 (PMC4423885; doi:10.1371/journal.pntd.0003739)
Supplement: S2 Table — (DOCX) [file pntd.0003739.s002.docx]

**Table S2. Within-host and population diversity in *P. falciparum* and *P. vivax* in active versus passively detected cases from Bangka and Sumba**

| **Island** | **Species** | **Detection** | **No. cases** | **% Polyclonal infections** | **Mean MOI (range)** | **Population diversity (mean *H*_E_)** |
| --- | --- | --- | --- | --- | --- | --- |
| Bangka | *P. falciparum* | Active | 22 | 9% (2/22) | 1.09 (1-2) | 0.437 |
|  |  | Passive | 36 | 17% (6/36) | 1.17 (1-2) | 0.435 |
|  |  |  |  | *P* = 0.697* | *P* = 0.429 | *P* = 1.000 |
| Bangka | *P. vivax* | Active | 37 | 35% (13/37) | 1.41 (1-3) | 0.837 |
|  |  | Passive | 49 | 45% (22/49) | 1.51 (1-3) | 0.847 |
|  |  |  |  | *P* = 0.490 | *P* = 0.389 | *P* = 0.860 |
| Sumba | *P. falciparum* | Active | 45 | 18% (8/45) | 1.22 (1-3) | 0.718 |
|  |  | Passive | 15 | 27% (4/15) | 1.27 (1-2) | 0.712 |
|  |  |  |  | *P* = 0.473* | *P* = 0.531 | *P* = 0.721 |

*Fisher’s exact test. Insufficient sample size in Sumba *P. vivax* passive case detection sample (*n*=9) for analysis.
