# Supplementary material for: Contrasting Transmission Dynamics of Co-endemic Plasmodium vivax and P. falciparum: Implications for Malaria Control and Elimination
Source: PLoS Negl Trop Dis. 2015 May 7;9(5):e0003739. doi: 10.1371/journal.pntd.0003739 (PMC4423885; doi:10.1371/journal.pntd.0003739)
Supplement: S5 Table — (DOCX) [file pntd.0003739.s005.docx]

**Table S5. Individual marker diversity in *P. falciparum* and *P. vivax***

| **Species** | **Marker** | **Bangka** | **Kalimantan** | **Sumba** | **Timor** |
| --- | --- | --- | --- | --- | --- |
| *P. falciparum* | Poly-alpha | 0.736 | 0.564 | 0.893 | 0.681 |
|  | ARAII | 0.165 | 0.538 | 0.822 | 0.637 |
|  | TA1 | 0.44 | 0.282 | 0.353 | 0 |
|  | TA87 | 0.629 | 0.462 | 0.663 | 0.481 |
|  | PfPK2 | 0.165 | 0.603 | 0.853 | 0.686 |
|  | TA81 | 0.537 | 0.731 | 0.810 | 0.650 |
|  | TA109 | 0 | 0 | 0.195 | 0 |
|  | TA42 | 0.457 | 0 | 0.490 | 0.611 |
|  | TA60 | 0.526 | 0 | 0.834 | 0.431 |
|  |  |  |  |  |  |
| *P. vivax* | pv3.27 | 0.906 | 0.974 | 0.958 | 0.833 |
|  | MS1 | 0.748 | 0.756 | 0.723 | 0.727 |
|  | MS10 | 0.896 | 0.936 | 0.896 | 0.741 |
|  | MS16 | 0.945 | 0.982 | 0.971 | 0.916 |
|  | MS5 | 0.872 | 0.861 | 0.874 | 0.835 |
|  | MS8 | 0.912 | 0.936 | 0.960 | 0.830 |
|  | MS12 | 0.814 | 0.621 | 0.814 | 0.804 |
|  | MS20 | 0.915 | 0.897 | 0.849 | 0.899 |
|  | msp1f3 | 0.596 | 0.692 | 0.660 | 0.670 |
