# Supplementary figures and images for: Contrasting Transmission Dynamics of Co-endemic Plasmodium vivax and P. falciparum: Implications for Malaria Control and Elimination
Source: PLoS Negl Trop Dis. 2015 May 7;9(5):e0003739. doi: 10.1371/journal.pntd.0003739 (PMC4423885; doi:10.1371/journal.pntd.0003739)

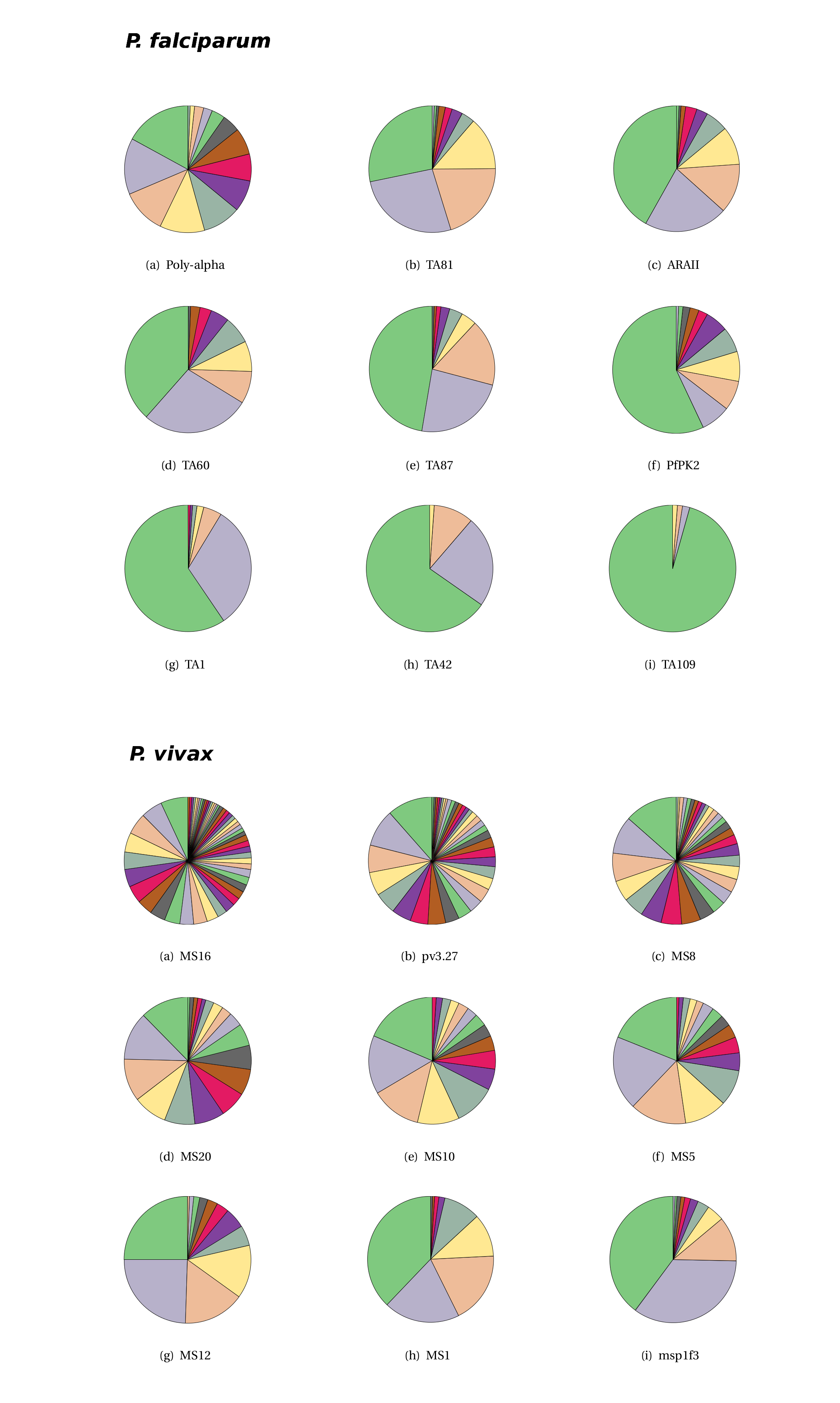

Supplement: S1 Fig — (TIF) [file pntd.0003739.s006.tif]

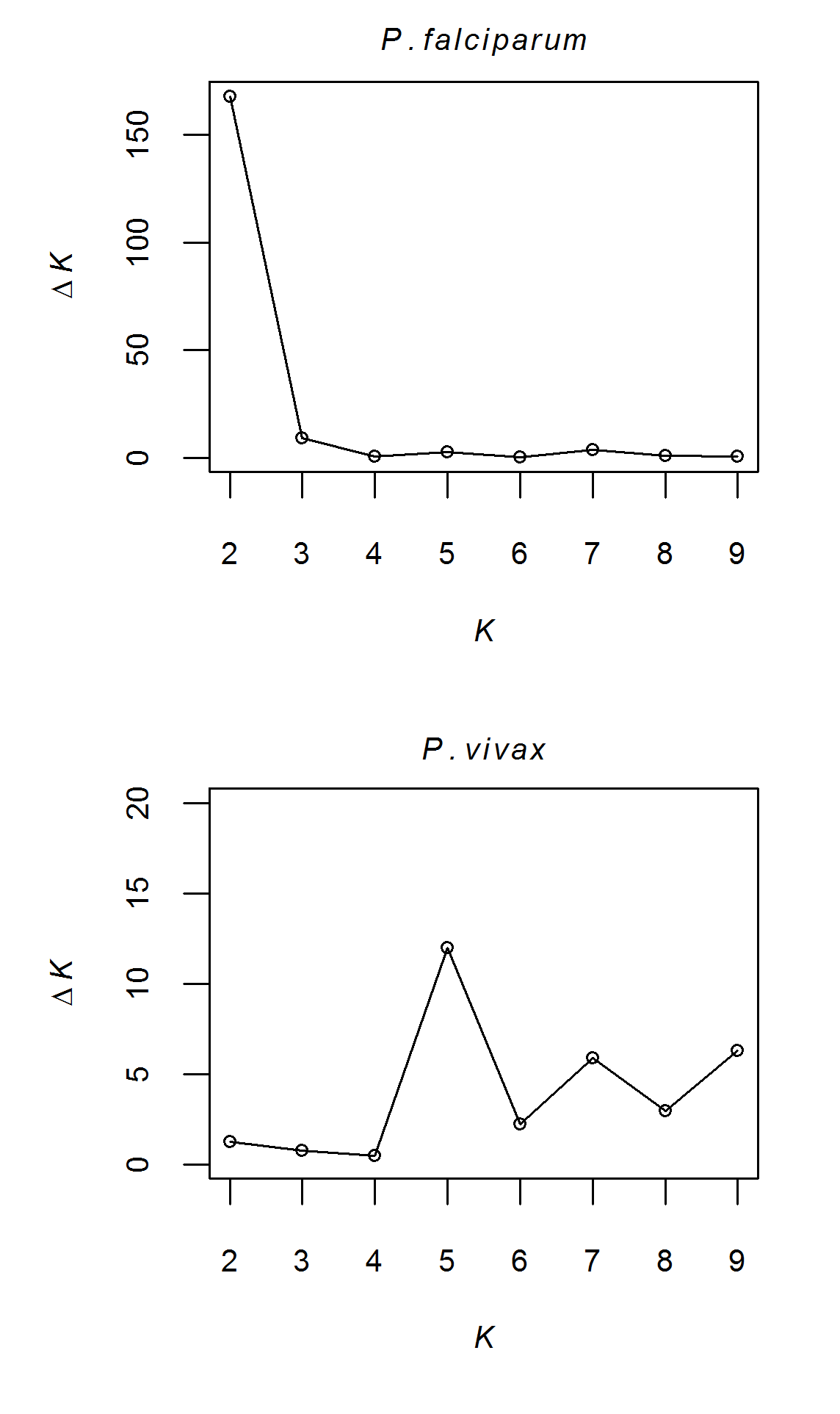

Supplement: S2 Fig — (TIFF) [file pntd.0003739.s007.tiff]
